# A comprehensive evolutionary scenario for the origin and neofunctionalization of the *Drosophila* speciation gene *Odysseus* (*OdsH*)

**DOI:** 10.1093/g3journal/jkad299

**Published:** 2023-12-29

**Authors:** William Vilas Boas Nunes, Daniel Siqueira Oliveira, Guilherme de Rezende Dias, Antonio Bernardo Carvalho, Ícaro Putinhon Caruso, Joice Matos Biselli, Nathalie Guegen, Abdou Akkouche, Nelly Burlet, Cristina Vieira, Claudia M A Carareto

**Affiliations:** Institute of Biosciences, Humanities and Exact Sciences, São Paulo State University (Unesp), 2265 Cristóvão Colombo Street, 15054-000 São José do Rio Preto, Brazil; Laboratoire de Biométrie et Biologie Evolutive UMR 5558, Université de Lyon, Université Lyon 1, CNRS, Bât. Grégor Mendel, 43 Boulevard 11 Novembre 1918, 69622 Villeurbanne, France; Institute of Biosciences, Humanities and Exact Sciences, São Paulo State University (Unesp), 2265 Cristóvão Colombo Street, 15054-000 São José do Rio Preto, Brazil; Laboratoire de Biométrie et Biologie Evolutive UMR 5558, Université de Lyon, Université Lyon 1, CNRS, Bât. Grégor Mendel, 43 Boulevard 11 Novembre 1918, 69622 Villeurbanne, France; Departamento de Genética, Instituto de Biologia, Universidade Federal do Rio de Janeiro, CCS sl A2-075, 373 Carlos Chagas Filho Avenue, 21941-971 Rio de Janeiro, Brazil; Departamento de Genética, Instituto de Biologia, Universidade Federal do Rio de Janeiro, CCS sl A2-075, 373 Carlos Chagas Filho Avenue, 21941-971 Rio de Janeiro, Brazil; Institute of Biosciences, Humanities and Exact Sciences, São Paulo State University (Unesp), 2265 Cristóvão Colombo Street, 15054-000 São José do Rio Preto, Brazil; Institute of Biosciences, Humanities and Exact Sciences, São Paulo State University (Unesp), 2265 Cristóvão Colombo Street, 15054-000 São José do Rio Preto, Brazil; Faculté de Médecine, iGReD, Université Clermont Auvergne, CNRS, INSERM, 4 Bd Claude Bernard, 63000 Clermont-Ferrande, France; Faculté de Médecine, iGReD, Université Clermont Auvergne, CNRS, INSERM, 4 Bd Claude Bernard, 63000 Clermont-Ferrande, France; Laboratoire de Biométrie et Biologie Evolutive UMR 5558, Université de Lyon, Université Lyon 1, CNRS, Bât. Grégor Mendel, 43 Boulevard 11 Novembre 1918, 69622 Villeurbanne, France; Laboratoire de Biométrie et Biologie Evolutive UMR 5558, Université de Lyon, Université Lyon 1, CNRS, Bât. Grégor Mendel, 43 Boulevard 11 Novembre 1918, 69622 Villeurbanne, France; Institute of Biosciences, Humanities and Exact Sciences, São Paulo State University (Unesp), 2265 Cristóvão Colombo Street, 15054-000 São José do Rio Preto, Brazil

**Keywords:** gene duplication, *unc-4*, homeodomain, transcription factor, *OdsH* expression, Drosophilidae

## Abstract

*Odysseus* (*OdsH*) was the first speciation gene described in *Drosophila* related to hybrid sterility in offspring of mating between *Drosophila mauritiana* and *Drosophila simulans*. Its origin is attributed to the duplication of the gene *unc-4* in the subgenus *Sophophora*. By using a much larger sample of Drosophilidae species, we showed that contrary to what has been previously proposed, *OdsH* origin occurred 62 MYA. Evolutionary rates, expression, and transcription factor–binding sites of *OdsH* evidence that it may have rapidly experienced neofunctionalization in male sexual functions. Furthermore, the analysis of the OdsH peptide allowed the identification of mutations of *D. mauritiana* that could result in incompatibility in hybrids. In order to find if *OdsH* could be related to hybrid sterility, beyond *Sophophora*, we explored the expression of *OdsH* in *Drosophila arizonae* and *Drosophila mojavensis*, a pair of sister species with incomplete reproductive isolation. Our data indicated that *OdsH* expression is not atypical in their male-sterile hybrids. In conclusion, we have proposed that the origin of *OdsH* occurred earlier than previously proposed, followed by neofunctionalization. Our results also suggested that its role as a speciation gene might be restricted to *D. mauritiana* and *D. simulans*.

## Introduction

*Odysseus* (*OdsH*) was the first so-called speciation gene characterized in *Drosophila*, specifically between *Drosophila mauritiana* and *Drosophila simulans* (Ting *et al*. 1998). The role of *OdsH* within the male hybrid sterility was attributed to the introgression of a sequence from *D. mauritiana* encompassing *OdsH* into the *D. simulans* genome (Perez *et al*. 1993; Perez and Wu 1995; Ting *et al*. 1998). The atypical expression of *OdsH* at the apical testis region was observed in these hybrids, which was not observed for fertile hybrids and parental species (Sun *et al*. 2004). The origin of the *OdsH* gene is proposed to have arisen by duplication of the *unc-4* gene, a conserved gene in Metazoa located *in tandem* with *OdsH* (Ting *et al*. 2004). The gene *OdsH*, the duplicated copy, is expressed in spermatocytes in species of the *melanogaster* subgroup and acts as a transcription factor binding to heterochromatic regions (Ting *et al*. 2004; Bayes and Malik 2009). Meanwhile, *unc-4*, the parental gene, is a transcription factor associated with motor neuron and proprioceptor developmental pathways in *Drosophila melanogaster* (Tabuchi *et al*. 1998; Lacin and Truman 2016; Lacin *et al*. 2019, 2020), similar to its conserved single-copy ortholog, which acts on motor neuron and optical sensorial cell development in *Caenorhabditis elegans* (Miller *et al*. 1992; Fox *et al*. 2005; Marques *et al*. 2019).

Both genes, *unc-4* and *OdsH*, encode homologous DNA-binding homeodomains, phylogenetically classified in the Paired-like class (Winnier *et al*. 1999; Copley 2005). The *OdsH* homeodomain has a high amino acid substitution rate in species of the *melanogaster* subgroup, corresponding to a higher divergence between the domains from *unc-4* between *Drosophila* and evolutionarily distant species, such as *C. elegans* (Ting *et al*. 2004). As expected for duplicated genes, the faster evolution of the *unc-4* paralog was associated with the acquisition of novel functions in the testis and with the speciation process (Ting *et al*. 1998, 2004).

Since the *OdsH* duplicate has been proposed to be a new gene in the *Sophophora* subgenus (Ting *et al*. 2004) and is associated with speciation in this clade, we would not expect to see this gene further in *Drosophila* phylogeny. However, searches in orthology databases GenTree (Shao *et al*. 2019) and OrthoDB (Kuznetsov *et al*. 2023) indicated the presence of *OdsH* duplicate in the ancestral node of the *Drosophila* genus, highlighting that its origin might be older than previously thought. We have thus asked the following questions: (1) how extensive is the presence of the *OdsH* duplicate in the *Drosophila* phylogeny; (2) did neofunctionalization in testis occur before the divergence of the *melanogaster* subgroup; and (3) is *OdsH* deregulation associated with the sterile hybrid phenotype in other recently diverged species, beyond the *D. melanogaster* group, such as those of the *mojavensis* complex (*repleta* group, subgenus *Drosophila*)? We showed that (1) the duplication occurred much earlier than previously proposed, dating back to 62 MYA in the Drosophilinae ancestor, (2) *OdsH* evolved under less intense negative selection than its paralog *unc-4* and has features that allow us to propose its ancient neofunctionalization in testis in the *Drosophila* genus, and (3) despite the presence and expression of *OdsH* in testis of the *Drosophila mojavensis* and *Drosophila arizonae*, no clear association was established between its deregulation with the observed hybrid sterility in the crosses between these species.

## Materials and methods

### *unc-4* and *OdsH* annotation in the Drosophilidae genomes

The sequences of *unc-4* and its duplicates were retrieved from publicly available annotated Drosophilidae genomes, focusing on its 2 sister subfamilies, Steganinae and Drosophilinae, with BLAST (NCBI), selecting the *High Scoring Pairs* (HSPs; Supplementary Table 1). The mRNA sequences with the highest *scores* and *e-values* smaller than 1−e05 were aligned with MAFFT (Katoh *et al*. 2002). The alignments were verified with BioEdit Sequence Alignment Editor v. 7.0.9 (Hall 1999) to remove the sequences that did not align and nonhomologous regions with *indels*. Therefore, the aligned sequences included a conserved region among the duplicates that contains the homeodomain (162 bp), with 15 bp upstream of the N-terminal homeodomain end and 228 bp downstream of the C-terminal end. The conserved region found in the *D. melanogaster* duplicates was used as a *query* with BLAST to search for homologous regions with available genome assemblies nonannotated on NCBI (Supplementary Table 2) using the same parameters as described for annotated genomes with a script in BASH language written by us. Sequences from *Chymomyza procmenis*, *Cacoxenus indagator*, and *Rhinoleucophenga bivisualis* were annotated in their genomes and assembled using SPADES v.3.9.0 software (Bankevich *et al*. 2012). For the annotation, the amino acid sequences of OdsH and Unc-4 of *D. melanogaster* were used as queries in TBLASTN searches in assembled genomes, and the scaffolds containing both homologous gene sequences were investigated on the coding sequences using the software GeneWise (Birney *et al*. 2004). Analysis of synteny was performed manually considering the Drosophilidae genomes available in the OrthoDB database (Kuznetsov *et al*. 2023). For *Scaptodrosophila lebanonensis* and *Leucophenga varia*, which are not available in OrthoDB, BLASTX (Altschul 1997) was used on the *D. melanogaster* protein database, considering a threshold of 70% of protein identity and coverage. In addition, we also looked for *unc-4* and possible duplicates in the publicly available genomes of Diptera, which are outgroups of Drosophilidae (Supplementary Table 3). Since we found only a single-copy duplication in these taxa, as in Steganinae, we decided to use only Steganinae data as the duplication outgroup for further analysis.

In order to identify possible gene losses in cases where the sequences were not found in assembled genomes, we performed the alignment of raw genome reads (*Drosophila erecta*: SRR22905006 and SRR22905007; *Drosophila ambigua*: SRR13070667; *Lordiphosa stackelbergi*: SRR13070699) against *unc-4* coding DNA sequence (CDS), using Bowtie 2 (Langmead and Salzberg 2012; Langmead *et al*. 2019 ): *Drosophila pseudoobscura* sequence for *D. ambigua*, *Lordiphosa collinella* for *L. stackelbergi*, and its own sequence for *D. erecta*. Thus, we aligned the unmapped reads against *OdsH* CDS: *D. pseudoobscura* sequence for *D. ambigua*, *L. collinella* for *L. stackelbergi*, and *Drosophila yakuba* for *D. erecta*. The alignments were visualized on Tablet (Milne *et al*. 2013).

### Phylogenetic inference and duplication dating

Phylogenetic relationships and dating were coestimated using the Bayesian molecular clock method and lognormal transformation to estimate the consensus tree topology and the divergence time (Drummond *et al*. 2006). It was possible to set the monophyly between *unc-4* and *OdsH* in Drosophilinae, as there is no evidence supporting duplicates *in tandem* in external taxa from such divergence. This method was used to avoid the phylogenetic bias *long branch attraction* (LBA; Felsenstein 1978; Hendy and Penny 1989), which has been demonstrated previously in phylogenetic heuristic methods with paralogs that have asymmetric evolution in *Drosophila* (Bao and Friedrich 2009). This method was used under the hypothesis that *unc-4* and *OdsH* evolved at different rates in comparison to the single-copy *unc-4* outgroup in Steganinae. Therefore, it could cause branch attraction in the most conserved gene, repulsion to the clade with the most divergent duplicate, and artifacts in the estimated dates.

Conserved region alignment was used to perform Bayesian inference of the phylogenetic relationships by the Yule process (Yule 1925; Gernhard 2008). For this, the software BEAST v. 1.6.1 (Drummond *et al*. 2006) was used with 5 categories of gamma distribution, invariable sites, and the substitution model GTR (Nei and Kumar 2000), estimated as the best substitution model by BIC on MEGA X (Kumar *et al*. 2018). The dating was carried out using the lognormal relaxed molecular clock (Drummond *et al*. 2006). The calibration was assessed using the estimated divergence from Suvorov *et al*. (2022) as the calibration points, as their report presents intermediate ages for Drosophilidae branches in comparison to previous studies: Drosophilidae family ancestor (63.19 MYA, 95% C.I.: 58.79–65.73 MYA), Drosophilini tribe ancestor (46.84 MYA, 95% C.I.: 43.85–49.85 MYA), and *D. melanogaster* × *D. simulans* divergence (3.62 MYA, 95% C.I.: 2.92–4.40 MYA) in the divergence node of its respective groups at the *unc-4* and *OdsH* clades. This calibration approach has been used to decrease the artifacts generated from the asymmetry in the substitution rates observed in the duplicates (Zhaxybayeva 2013). The inference was carried out using the Markov chain Monte Carlo (MCMC) model with 10,000 samples in each 1,000 chains (Drummond *et al*. 2012). Subsequently, the first 1,000 samples were removed with the *burn-in* option in TreeAnnotator (Drummond *et al*. 2006), and then the estimated consensus tree was created with the best posterior probability (PP) for each node. The tree was visualized and customized with FigTree 1.4 (Rambaut 2009).

### Codon usage bias

Taking into account that codon usage bias may result in phylogenetic artifacts in gene trees (Inagaki *et al*. 2004; Inagaki and Roger 2006; Liu *et al*. 2014), due to differences in codon usage in the *saltans* and *willistoni* radiations in comparison to other *Drosophila* groups (Powell *et al*. 2003; Vicario *et al*. 2007), and because the *Drosophila willistoni* phylogenetic position is commonly an artifact (Pélandakis and Solignac 1993; Gailey *et al*. 2000; Tarrío *et al*. 2001), the analyses were performed to estimate the *relative synonymous codon usage* (RSCU) by group and by gene. The RSCU was carried out with MEGA X (Kumar *et al*. 2018), along with CAIcal (Puigbò *et al*. 2008), to identify the effective number of codons (ENC) and the GC proportion at the third codon position (%GC3). We carried out a principal component analysis (PCA) to investigate the difference between the RSCU of Drosophilidae groups and a *t* test to verify the difference between the ENC and %GC3 between the clade *willistoni*–*saltans*–*Lordiphosa* and the rest of the Drosophilidae phylogeny. The statistical analyses were conducted in R v. 4.1.2 (R Core Team 2021).

### Relative rate of nucleotide substitution

To identify whether *unc-4* and its duplicates are evolving at different rates, the relative rate test was performed with PHYLTEST 2.0 (Kumar 1996). The external groups used were the *unc-4* sequences annotated from Steganinae species, applying Kimura 2-parameters (Kimura 1980) as the best substitution model.

### Estimates of selective pressure and investigation of signatures of positive selection

To characterize the selection acting on the *unc-4* and *OdsH* genes, codon-based likelihood methods were run using the CODEML package in PAML version 4.9 (Yang 2007). Maximum likelihood estimates of the selective pressure were measured by the nucleotide substitution rate (*ω* = *K*_a_/*K*_s_) of nonsynonymous (*K*_a_) to synonymous (*K*_s_) substitutions. For these analyses, 2 trees in Newick format were used, 1 of which was *Tree 1*, described above, using the alignment of the sequences *unc-4* and *OdsH*. Since only the *OdsH* sequences presented nonsynonymous substitutions, selection tests were also performed considering only this gene, constructing a tree, hereafter referred to as *Tree 2*, also by Bayesian inference, with the same priors as for *Tree 1*. For these analyses, the branch model test allows the *ω* ratio to vary among branches in the phylogeny (Yang 1998; Yang and Nielsen 1998). This approach was applied to estimate the *ω*-value in *Tree 1*, with labels in *unc-4* and *OdsH* nodes, and in *Tree 2*, labeling each group of species. The same labels were applied for the test of relaxation of the strength of natural selection through RELAX (Wertheim *et al*. 2015), implemented in HyPhy (Pond *et al*. 2005) to identify possible relaxation of selective constraints in the *OdsH* branch. In addition to that, codeml was used to test the site model in order to identify signatures of positive selection by sites of each group of species. All the hypotheses developed to identify the *ω*-value were tested using the *χ*^2^ test, with the comparison of the lnL values of each hypothesis.

### Transcription factor–binding sites at the *unc-4* and *OdsH* regulatory regions

To investigate the presence of different transcription factor–binding sites (TFBSs) located at the *unc-4* and *OdsH* regulatory regions, the sequences were extracted 500 bp upstream and downstream of the genes from all species in which expression could be analyzed in silico (described below; Supplementary Table 4). In addition, the sequences of *Drosophila sechellia*, *D. simulans*, and *D. mauritiana* were included because in these species, *OdsH* is associated with hybrid sterility, and of *D. arizonae*, present in our database, as it can cross and produce sterile offspring with *D. mojavensis* (Supplementary Table 4). For this analysis, the *OdsH* regulatory sequences were subjected to enrichment analysis with CiiiDER (Gearing *et al*. 2019) to identify differentially enriched TFBSs between *unc-4* and *OdsH* by using the *unc-4* sequences as background. We used the JASPAR CORE (Castro-Mondragon *et al*. 2022) database of insect TFBSs for this analysis. The *deficit threshold* default (0.15) and the Fisher *P*-value threshold 0.05 were applied. The transcription factors with differential enrichment of binding sites to the regulatory regions between *unc-4* and *OdsH* were used for Gene Ontology (GO) analysis (Ashburner *et al*. 2000; Mi *et al*. 2019) in the biological process category.

### Protein functional motifs

The homeodomains and the octapeptide were found in Unc-4 and OdsH proteins separately with MEME (Bailey and Elkan 1994) in the MEME Suite platform (Bailey *et al*. 2015). To observe the wide pattern of homeodomain diversity in both proteins from Drosophilinae, they were calculated with the translated sequences retrieved from the Drosophilinae alignment. The octapeptide was estimated from the alignment of the 11 C-terminal amino acids of the Unc-4 and OdsH proteins, as reported in NCBI (Supplementary Table 1).

The binding stability of the tridimensional models for the Unc-4 and OdsH homeodomains associated with the DNA was assessed through in silico investigation to infer whether their protein sequence divergence could cause functional divergence. The protein modeling of the Unc-4 and OdsH homeodomains was developed with SWISS-MODEL (Waterhouse *et al*. 2018) using the structure of PDB 3LNQ (Miyazono *et al*. 2010) as a template. The modeling was performed for *D. melanogaster* (NP_573242.2 and NP_523389.3) and for *Teleopsis dalmani* Unc-4 (XP_037943702.1) as an outgroup to the duplication event. Afterwards, the complexes derived from the structural model Unc-4 from *T. dalmani* and the DNA structure were minimized from molecular dynamic simulations using GROMACS (Abraham *et al*. 2015), applying the AMBER14-OL15 package with ff14sb protein (Maier *et al*. 2015) and ff99bsc0OL15 DNA (Zgarbová *et al*. 2015) force fields, as well as the TIP3P1 water model (Jorgensen *et al*. 1983).

The simulated molecular system was inserted into a solvated cubical box with a 100 mM NaCl solution in water. Energy minimization was performed with the *steepest descent integrator* and the conjugated gradient algorithm, with 500 kJ/mol/nm, as the maximum force threshold. The calculation of the perturbation values of the variation in the free energy of ligation (ΔΔGb) was assessed with the observed OdsH substitutions in *Drosophila*, which interferes with the stability of the homeodomain/DNA complex, by using the mCSM server (Pires *et al*. 2014), in comparison to the Unc-4/DNA homeodomain complex structure.

### *OdsH* and *unc-4* expression

To answer the question of whether is *OdsH* associated to hybrid sterility outside of the *D. melanogaster* group, the expression profiles of *unc-4* and *OdsH* were manually inspected with the *Tracks* tool from the *Gene* platform available at NCBI (www.ncbi.nlm.nih.gov/gene) using public databases. All Drosophilinae species with available transcriptome expression data from either reproductive or nonreproductive tissues were analyzed separately by sex (Supplementary Table 4). The same approach was used to identify the expression of the single-copy *unc-4* gene in the *T. dalmani* genome as an outgroup to the duplication event. For each species and tissue, the genes were characterized as expressed when they had >10 *counts* identified at the expression histogram from the *Tracks* tool.

Experimental analysis of *OdsH* expression in hybrids was conducted in *D. mojavensis baja* and *D. arizonae* and their offspring, which produce fertile and sterile hybrids in the laboratory depending on the strain and direction of crossing. For this, intra- and interspecific crosses were performed in both directions between *D. arizonae* from Metztitlan, Hidalgo, Mexico (Stock Center n.: 15081–1271.**17**), and *D. mojavensis baja* from the Cape Region, Santiago, Baja California Sur, Mexico (Stock Center n.: 15081–1352.**20**). These species were chosen as representatives of the *Drosophila* subgenus, allowing the observation of *OdsH* functions outside the *Sophophora* subgenus previously reported. In addition, they show recent divergence and incomplete reproductive isolation. Their reciprocal interspecific crosses are asymmetrical, with the male offspring being fertile when descended from male *D. arizonae* (H♀moj^baja^♂ari) and sterile when descended from male *D. mojavensis baja* (H♀ari♂moj^baja^) and the female offspring being fertile in both directions (Banho *et al*. 2021). Besides that, their sterile hybrids present a phenotype with defective sperm bundles (Hardy *et al*. 2011; Kanippayoor *et al*. 2020), similar to the sterile offspring from *D. mauritiana* and *D. simulans* (Perez *et al*. 1993). Since deregulation in hybrids might result from fast male evolution, the comparison between fertile and sterile hybrids can help to determine specific deregulation related to sterility (Gomes and Civetta 2014, 2015).

For the experimental crosses, virgin males and females were collected until 48 h after emergence and isolated in tubes containing *Opuntia* sp.–based media for 3 days. For this, each cross was performed in 35 replicates, each containing 10 couples, for 12 days. The testes of descendants (10–12 days) were dissected in 1× PBS. Dissected testes in 1× PBS from both hybrids and parental species were subjected to smRNA FISH to determine if *OdsH* had atypical expression in sterile hybrids, considering the spermatogenesis phases. The testes were then fixed in fixing buffer (4% formaldehyde, 0.3% Triton X-100, and 1× PBS) for 20 min at room temperature, rinsed 3 times in 0.3% Triton X-10, 1 in PBS, and permeabilized in 70% ethanol at 4°C overnight. Permeabilized testes were rehydrated in smRNA FISH wash buffer (10% formamide in 2× SSC) for 10 min. Testes were resuspended in 50 μL hybridization buffer (10% dextran sulfate, 10% formamide in 2× SSC, supplemented with 1 μL of smRNA FISH probes) designed with Stellaris Probe Designer version 4.2 (https://www.biosearchtech.com/stellaris-designer; Supplementary Table 5), synthesized, and labeled with ATTO 550. Hybridization was performed with rotation at 37°C overnight. Testes were then washed twice with smRNA FISH wash buffer at 37°C for 30 min and twice with 2× SSC solution. Then, DNA was stained with DAPI (Thermo Fisher Scientific; 1/500 dilution in 2× SSC) at room temperature for 20 min. Images were captured using an upright Zeiss LSM780-NLO confocal microscope.

For quantitative analysis, the RNA was extracted from the testes of 7 biological replicates each using 25 individuals using the RNeasy kit (Qiagen) and was treated with DNase (DNA-free kit; Ambion). For each replicate, 1,000 ng of RNA was converted to cDNA using a *High Capacity cDNA Reverse Transcription kit* (Thermo Fisher). The relative level of mRNA was quantified using specific oligonucleotides and probes (TaqMan, Thermo Fisher Scientific) for *OdsH* (forward primer: AGCCGCAGAGCTGCA; reverse primer: GCTCGATCGCCTTGGCTAT; probe: CTGCAGGAGCTGCGAGCCA). qPCR was then conducted using 3 technical replicates, each containing 100 ng of cDNA in a *LightCycler* 480 (Roche Diagnostics). The expression level was measured by the relative quantification (RQ) ratio in relation to the *endogenous ribosomal gene 49* (*rp49*), also known as nrpL (forward primer: CCCAACATTGGTTACGGTTCCA; reverse primer: GCACATTGTGTACGAGGAATTTCTT; probe: CACCCGCCACATGCT). Then, the relative quantity of the transcripts was normalized by the following expression: (RQ = *E*^Ct rp49^/*E*^Ct OdsH^; *E* = reaction efficiency). The normalized values were subjected to Shapiro–Wilk and Bartlett tests for each tissue. Since they did not present a normal distribution and variance homogeneity, their variances were calculated through the Kruskal–Wallis test.

## Results

### How extensive is the presence of the *OdsH* duplicate in the *Drosophila* phylogeny?

#### Occurrence and phylogenetic relationships

**Fig. 1.**
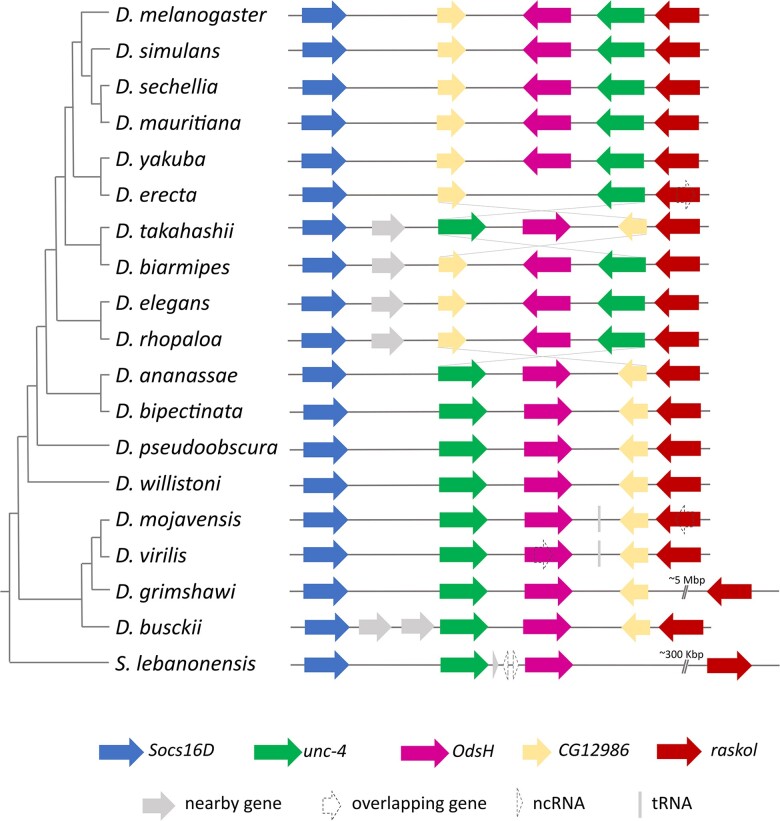
Relative positions of gene sequences in the neighborhood of *OdsH* and *unc-4* in Drosophilinae genomes. The representation of the phylogenetic relationships is based on Suvorov *et al*. (2022).

The search for sequences of the *unc-4* gene and its duplicate in annotated genomes (36 species) found them adjacently placed in the genomes of all species, and the synteny was conserved in their genomic neighborhood (encompassing *Socs16D*, CG12986, and *raskol* genes; Fig. 1) along the *Drosophila* phylogeny. Exceptionally, the genome assembly of *D. erecta* lacked any evidence of the duplicate and presented no genomic read that aligned to *OdsH*. We also observed that the genomic fragment formed by the sequences of the genes *unc-4*, *OdsH*, and *CG12896* probably underwent an inversion in the *melanogaster* subgroup ancestor and in *Drosophila takahashii* (Fig. 1). The investigated genomes from the subfamily Steganinae (4) returned only the *unc-4* sequence (Supplementary Table 2). In *L. varia*, the only Steganinae representative that has genome assembled in contigs, the sequences of the reference neighbor genes (*Socs16D* and *raskol*) were found very far from the single-copy *unc-4* sequence (*raskol* at 1.8 million base pairs and *Socs16D* at 4 million, both upstream), being its neighbors *CG17209* upstream and *CG14213* downstream. No evidence of *unc-4* duplicates was found in genomes of the non-Drosophilidae Diptera (Supplementary Table 3).

**Fig. 2.**
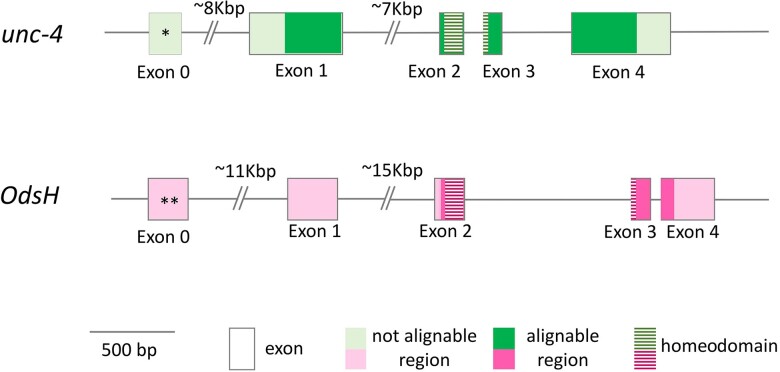
Gene structure of *unc-4* and *OdsH* in Drosophilinae. An asterisk denotes present only in *D. ananassae*, *D. virilis*, and *D. grimshawi*. A double asterisk denotes present only in *D. arizonae*, *D. mojavensis*, and *S. lebanonensis*.

The distances between the duplicates varied between 10,982 bp (*D. simulans*) and 80,454 bp (*S. lebanonensis*) and were 31,393 bp on average. The lengths of the *OdsH* genes ranged between 5,195 bp (*Drosophila busckii*) and 37,364 bp (*D. willistoni*), with an average of 23,027 bp. The lengths of *unc-4* ranged between 7,801 bp (*D. willistoni*) and 21,691 bp (*Drosophila virilis*), with an average of 11,536 bp. Although both genes present a general structure containing 4 exons (Fig. 2), they differ in size, mainly due to the longer introns in *OdsH*. Additionally, there is no signal of homology between their exon 1. Furthermore, *D. mojavensis*, *D. arizonae*, and *S. lebanonensis* showed an extra exon upstream of the *OdsH* first exon, here referred to as exon 0. The same was observed for *unc-4* of *Drosophila ananassae*, *D. virilis*, and *Drosophila grimshawi.* These extra exons probably arose independently in different evolutionary lineages since they show no homology among the orthologs from different groups (Fig. 2).

Sequences homologous to both duplicates were also searched Drosophilinae nonannotated genomes (101 species; Supplementary Table 2) and were identified in all of them, except for *D. ambigua* that presented no sequences for both duplicates and *L. stackelbergi* that presented only *unc-4* homologous sequence. However, *D. ambigua* and *L. stackelbergi* presented genomic reads aligning to both genes (Supplementary Fig. 1), evidencing that these species genomes most likely lack *OdsH* because it was not assembled.

All *unc-4* and *OdsH* sequences that had all their exons within the same scaffold were used to infer the phylogenetic relationships between the 2 genes, and they segregated into 2 sister monophyletic groups, supporting the hypothesis of orthology between the obtained *OdsH* sequences and the predicted *OdsH* of *D. melanogaster* and *D. sechellia*, as well as the paralogy in relation to *unc-4* (Supplementary Fig. 2). Although the sequences of the *willistoni*–*saltans*–*Lordiphosa* radiation, which form a robust monophyletic cluster, coalesce to the common ancestral nodes in both the *unc-4* and *OdsH* clades, their positioning in both clades is inconsistent with the evolutionary history of Drosophilinae. This radiation grouped at the bottom of the Drosophilini branch for both genes. This incongruity may be due to the differential use of codons in this lineage in relation to the others, as already reported for the species of the groups *willistoni* and *saltans* (Rodríguez-Trelles *et al*. 2000; Singh *et al*. 2006; Vicario *et al*. 2007). We then calculated the RSCU, the ENC, and the %GC3. The PCA of the RSCU data showed different codon usage patterns for *unc-4* and *OdsH* among species. For both genes, the *willistoni* and *saltans* groups, as well as the single-copy *unc-4* of the Steganinae subfamily, were clustered with ∼37% variance from the *Drosophila* subgenus (Supplementary Figs. 3 and 4). In addition, higher ENC values and lower %GC3 were observed in *unc-4* sequences from the *willistoni*–*saltans*–*Lordiphosa* branch in comparison to the other Drosophilini (ENC: *t* = −4.27, *P* = 3e^–05^; %GC3: *t* = 9.335, *P* < 0.00001; Supplementary Fig. 5) and in *OdsH* (ENC: *t* = −4.677, *P* ≤ 0.00001; %GC3: *t* = 9.884, *P* < 0.00001; Supplementary Fig. 6). Knowing that differences in the use of codons can cause phylogenetic artifacts (Inagaki *et al*. 2004; Inagaki and Roger 2006; Liu *et al*. 2014), we removed these sequences from the phylogenetic analyses. In addition, sequences from groups of species that were clustered incongruently in the phylogeny in relation to the *Drosophila* subgenera were also removed to avoid biases in the analyses of duplication dating and selection.

**Fig. 3.**
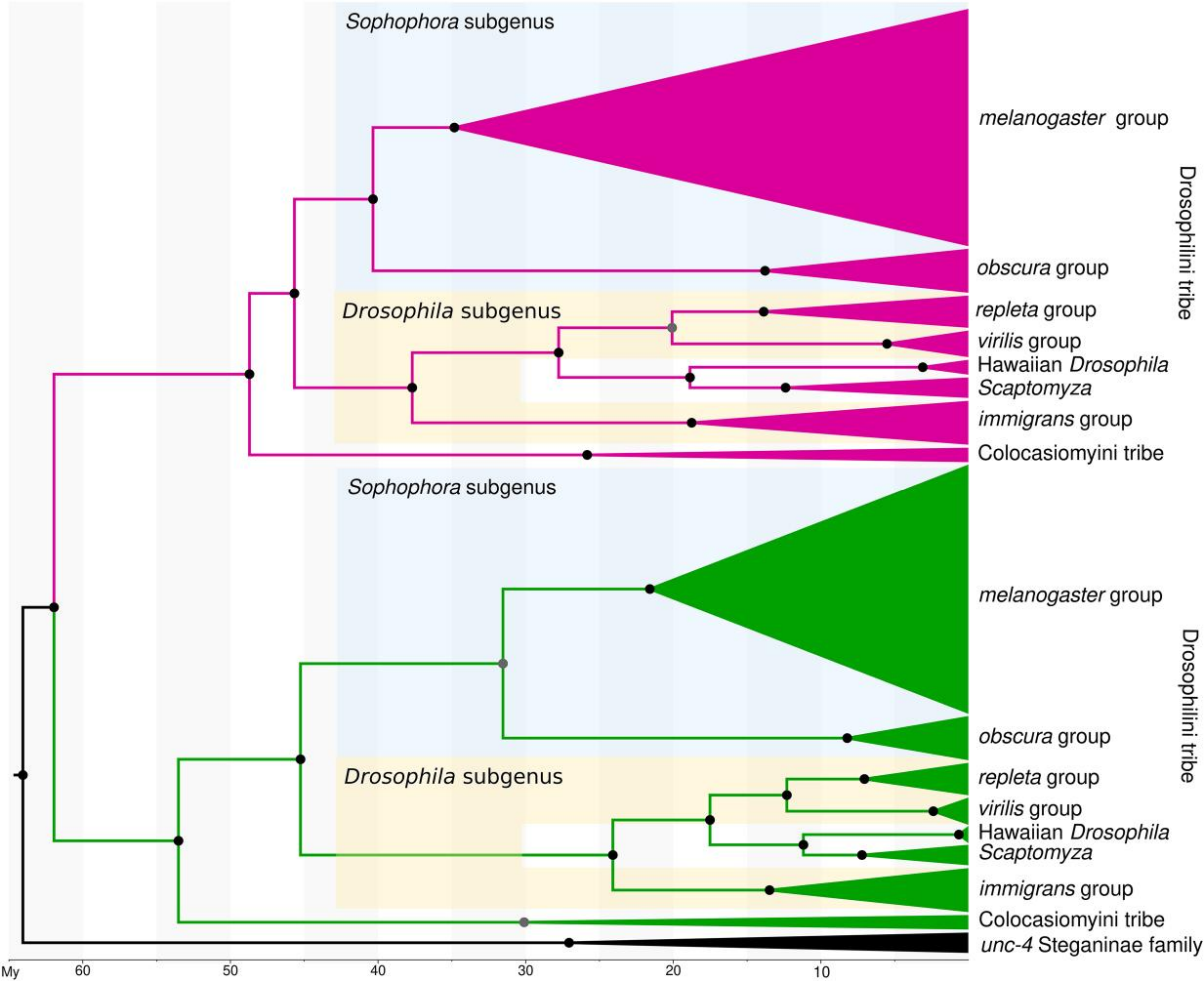
Calibrated Bayesian phylogenetic inference of the sequences of the paralog genes *unc-4* and *OdsH* using the GTR+G+I substitution model. The analysis was performed with 405 nucleotide sites from 162 sequences. All positions containing gaps and ambiguous bases were removed from the pairwise sequence analysis. The branches referring to the *Drosophila* taxonomic groups were compressed. At the root of each clade, the PP is presented by black (darker) (>0.9) and gray (lighter) (>0.7) circles, and the estimated times of divergence are indicated. The analysis was conducted in BEAST v16.1. The *unc-4* clade (green), subdivided into the more basal single-copy *Steganinae* (outgroup—black) and Drosophilinae, is presented at the base of the phylogeny followed by the *OdsH* clade in the upper part (pink). Monophyletic taxonomic groups of the *Drosophila* genus were compressed. Uncompressed clades can be seen in Supplementary Fig. 7. Subgenera are highlighted in blue (*Sophophora*) and yellow (*Drosophila*).

We used Bayesian inference to estimate the tree topology and the divergence time between *unc-4* and *OdsH* sequences of Drosophilinae. The monophyly of these genes was confirmed, building sister clades generally comprising the subgenera and species groups of Drosophilinae (Fig. 3; Supplementary Fig. 7). The node shared by these 2 clades, which represents the duplication event, rooted by the *unc-4* single-copy sequences of the Steganinae clade, dated back to 62 MYA. The *OdsH* clade has longer branches than *unc-4*, with older ages for the nodes of the taxa, an artifact due to the greater divergence between its sequences than between those of *unc-4*. However, the clades *OdsH* and *unc-4* show congruence regarding the monophyly of the tribes Drosophilini and Colocasiomyini and of the subgenus *Sophophora*, positioned basally in the tribe Drosophilini.

### Did neofunctionalization in testis occur before the divergence of the *melanogaster* subgroup?

#### Evolutionary dynamics

In order to identify if the coding sequence of *OdsH* evolved faster than *unc-4*, we looked for the comparison of evolutionary rates for these genes, since faster evolution could be an evidence of neofunctionalization (Van de Peer *et al*. 2001; Jordan *et al*. 2004; Dong *et al*. 2012; Pegueroles *et al*. 2013; Chakraborty and Fry 2015). The rate of nucleotide substitution was higher in *OdsH* than in *unc-4* (*Z* = 8.395, *P* < 0.05) in relation to the *unc-4* single copy of the outgroup. The signatures of selection on *OdsH* were estimated by the branch model—model 2 (2 ratio) by labeling each gene, using the tree estimated for them (*Tree 1*), and for each group of species represented by more than 3 sequences, with a tree estimated using only *OdsH* sequences (*Tree 2*). Negative selection was predominantly observed in the evolution of the 2 genes (*ω* < 1) in the branch model 2 analysis; however, the mean values of *ω* differed significantly (*χ*^2^ = 50.678, *P* = 9.894e^–12^), being more than 5 times higher for *OdsH* (*ω* = 0.194) than for *unc-4* (*ω* = 0.037), when considering the *OdsH* ancestor node. Regarding *OdsH* divergence along Drosophilinae tree, *ω*-value was lower than the ancestor node (*ω* = 0.04196) but still higher than *unc-4* (*ω* = 0.01545, *χ*^2^ = 48.589, *P* = 2.817e^–11^). A single nonsynonymous substitution in *unc-4* was observed in the outgroup *R. bivisualis* (T118Q). In the test for relaxation of negative selection, *OdsH* presented signatures of relaxed selection in comparison to *unc-4* (*K* = 0.08; *P* = 0). However, no signals of sites under positive selection in *OdsH* were detected along the complete Drosophilinae branch (*χ*^2^ = 0.003, *P* = 0.999).

As no nonsynonymous substitution was observed in the Drosophilinae *unc-4* sequences, branch model analysis was not performed for this gene considering each Drosophilinae group. For the selection acting on *OdsH*, no differences were observed between the groups of Drosophilinae species (Table 1), except for sequences of the *D. melanogaster* complex (*ω* = 0.320, *χ*^2^ = 39.047, *P* < 4.14e^–10^) and *immigrans* group (*ω* = 0.047, *χ*^2^ = 5.855, *P* = 0.016). The *immigrans* group higher *ω* can be explained by signatures of positive selection (*ω* = 3.626, *χ*^2^ = 7.258, *P* = 0.027; Supplementary Table 6). Meanwhile, the *melanogaster* complex presented no evidence of positive selection, being its divergence most likely has been driven by relaxation of negative selection (*K* = 0.16, *P* = 0).

**Table 1.** Selective process acting on *OdsH* in branches of Drosophilinae.

Taxon	Branch test		Site test		Relaxation test
	*ω*	*χ* ^2^ (*P*-value)	*ω*	*χ* ^2^ (*P*-value)	*K* (*P*-value)
*melanogaster* group	0.046	3.766 (0.052)	2.932	0.001 (0.999)	1.52 (0.536)
*melanogaster* complex	**0**.**320**	**39.047** (**0.000)**	8.023	5.824 (0.054)	**0.16** (**0.000)**
*obscura* group	0.020	2.903 (0.088)	2.846	0.002 (0.999)	9.73 (0.131)
*repleta* group	0.0001	0.409 (0.522)	1.338	0.349 (0.840)	0.92 (0.685)
*virilis* groups	0.037	0.006 (0.940)	1	0.000 (1.000)	1.39 (0.457)
Hawaiian *Drosophila*	0.118	2.568 (0.109)	1	0.002 (0.999)	10.47 (0.131)
*Scaptomyza*	0.017	2.094 (0.148)	1	0.001 (1.000)	0.20 (0.055)
*immigrans* group	**0**.**047**	**5.855** (**0.016)**	**3.626**	**7.258** (**0.027)**	1.07 (0.694)
Colocasiomyini	0.007	0.170 (0.680)	1	0.001 (0.999)	1.13 (0.413)
H0	0.039	—	—	—	—

Groups that have significantly different values are highlighted in bold (*P* < 0.05).

H0, null hypothesis.

#### Candidate regulators of *OdsH* and *unc-4* expression

**Fig. 4.**
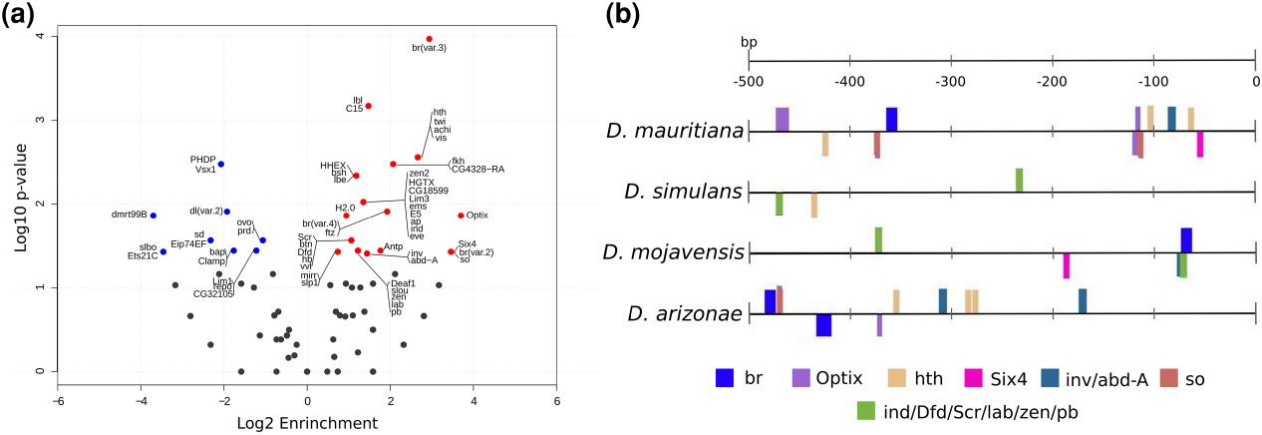
Enriched TFBSs in the regulatory sequence of *unc-4* and *OdsH.* a) TFBS enrichment values for *OdsH* (red) and *unc-4* (blue). Gray dots represent transcription factors whose binding sites did not differ from each other. b) Differentially present enriched TFBS between sister hybridizing species.

The comparison of the 500 bp upstream and downstream regions of *OdsH* and *unc-4* showed that *OdsH* was enriched for 43 and 15 TFBSs, respectively, while *unc-4* upstream and downstream regions had 15 and 13 TFBSs, respectively (Fig. 4a; Supplementary Tables 7 and 8). Transcription factors that putatively bind to the regulatory region of *OdsH* showed a wide diversity of GO categories primarily related to development and organogenesis, while those of *unc-4* were also related to leg development and morphogenesis (Supplementary Fig. 8). In the upstream region of *OdsH*, the enrichment of TBFSs attributed to the category of development process involved in male reproduction stood out, specifically *achi*, *vis*, and *so*, which are related to the spermatogenesis category.

Among the enriched TFBSs, we have looked for their presence in *D. mauritiana* and *D. simulans*, as well as, in *D. mojavensis* and *D. arizonae*, in order to identify potential regulatory divergence between these species pair that could possibly cause *OdsH* deregulation in hybrids. We found 13 transcription factors that had binding sites present in 1 species but not in its sister species (Fig. 4b) and among them, 12 contain homeodomain motifs (all of them, except for *br*).

#### Functional protein motifs

**Fig. 5.**
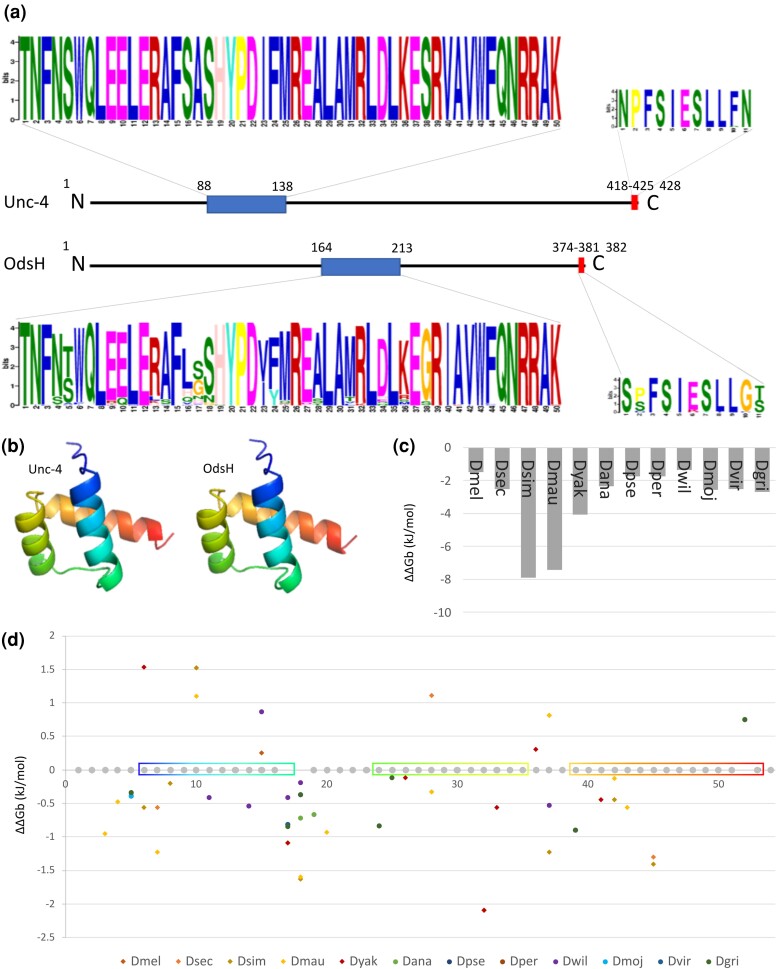
Functional motifs in Unc-4 and OdsH proteins. a) Representations of the Unc-4 and OdsH primary structures in *D. melanogaster* and functional motifs found in Drosophilinae: homeodomain (blue) and octapeptide (red). b) 3D models of Unc-4 and OdsH homeodomains. The N-terminal tail is presented in blue, and the C-terminal tail is presented in red. c) Total energy variation of the OdsH and DNA homeodomain complex, by species, in relation to Unc-4. d) Energy variation of the OdsH and DNA homeodomain complex, per substitution, relative to Unc-4, by species along the amino acid chain (0–54). Sites without a gray circle represent replacement in all analyzed species. The boxes represent the positions of the 3 α-helices. Overlapping dots represent shared mutations: 5—*D. mojavensis*, *D. virilis*, and *D. grimshawi*; 5—*D. persimilis* and *D. pseudoobscura*; 17—*melanogaster* complex; 17—*D. persimilis*, *D. pseudoobscura*, *D. ananassae*, *D. mojavensis*, and *D. virilis*; 18—*D. sechellia* and *D. simulans*; 18—*D. persimilis*, *D. pseudoobscura*, *D. mojavensis*, *D. virilis*, and *D. grimshawi*; 19—*D. simulans*, *D. mauritiana*, *D. yakuba*, and *D. ananassae*; 32—*melanogaster* complex; 37—*D. melanogaster*, *D. sechellia*, and *D. mauritiana*; 39—all except *D. simulans*, *D. sechellia*, and *D. mauritiana*; and 52—all species.

The homeodomain and the C-terminal octapeptide were conserved in the sequences of the Unc-4 proteins (*e*-values—homeodomain: 1.0e^–3418^; octapeptide: 5.8e^–238^) and OdsH (*e*-values—homeodomain: 2.3e^–3505^; octapeptide: 1.6e^−189^) in *Drosophila*, as seen in the scheme of primary structures in *D. melanogaster* (Fig. 5). In both motifs, there was greater divergence in OdsH, while the Unc-4 motifs did not show amino acid substitutions (Fig. 5a). The OdsH octapeptide has a core of 8 conserved amino acids, and the adjacent amino acids exhibit some divergence. OdsH in *D. mauritiana* is missing the octapeptide, since there is a truncation at the C-terminal region. The 3D models of the homeodomains showed the usual secondary structure of 3 alpha helices with an N-terminal tail in a segment of 54 amino acid residues (Fig. 5b), with the exception of amino acid 53 at the C-terminal end of the third helix in OdsH. In Unc-4, this amino acid does not participate in the structure. Since the Unc-4 homeodomain did not have substitutions in *Drosophila* or in *T. dalmani*, there was no variation in the free energy of protein/DNA binding (ΔΔGb). Conversely, OdsH homeodomains showed higher DNA binding instability, which was more pronounced in *D. simulans* (−7,896 kJ/mol) and *D. mauritiana* (−7,414 kJ/mol; Fig. 5c). Most OdsH homeodomain substitutions destabilized the complex with DNA (ΔΔGb < 0; Fig. 5d). It was generally observed that the species had different substitutions in OdsH that resulted in different ΔΔGb per site, except for *Drosophila persimilis* and *D. pseudoobscura*, which have identical sequences, and *D. mojavensis*, *D. virilis*, and *D. grimshawi*, which have similar numbers of amino acid substitutions (6 substitutions in *D. mojavensis* and *D. virilis* and 7 in *D. grimshawi*, 4 of which were shared between the 3 species). The species in the *melanogaster* subgroup had substitutions that resulted in the highest ΔΔGb values. A greater number of substitutions were found in the first α-helix. In the third α-helix, which makes direct contact with the DNA, there were 2 substitutions shared by different groups (S40G, except for *D. simulans*, *D. mauritiana*, and *D. sechellia*, which shared the ancestral allele, and V53W). The other substitutions in this helix were species specific and were present exclusively in the *melanogaster* group.

### Is the deregulation of *OdsH* expression in the testis associated with sterility of hybrid males beyond the *D. melanogaster* group?

#### Expression of *OdsH* and unc-4 in *D. arizonae*, *D. mojavensis baja*, and their hybrids

The analysis of the *Drosophila* transcriptomes available in public databases (*D. pseudoobscura*: PRJNA291085; *D. grimshawi*: PRJNA317989; *T. dalmani*: PRJNA240197; other species: PRJNA388952) showed that both genes have low expression levels. However, *unc-4* seems to be mainly expressed in somatic tissues, whereas *OdsH* seems to be specific to male reproductive tissues (Supplementary Fig. 9). This is expected in the cases of neofunctionalization, suggesting that *OdsH* neofunctionalization occurred rapidly after its origin.

**Fig. 6.**
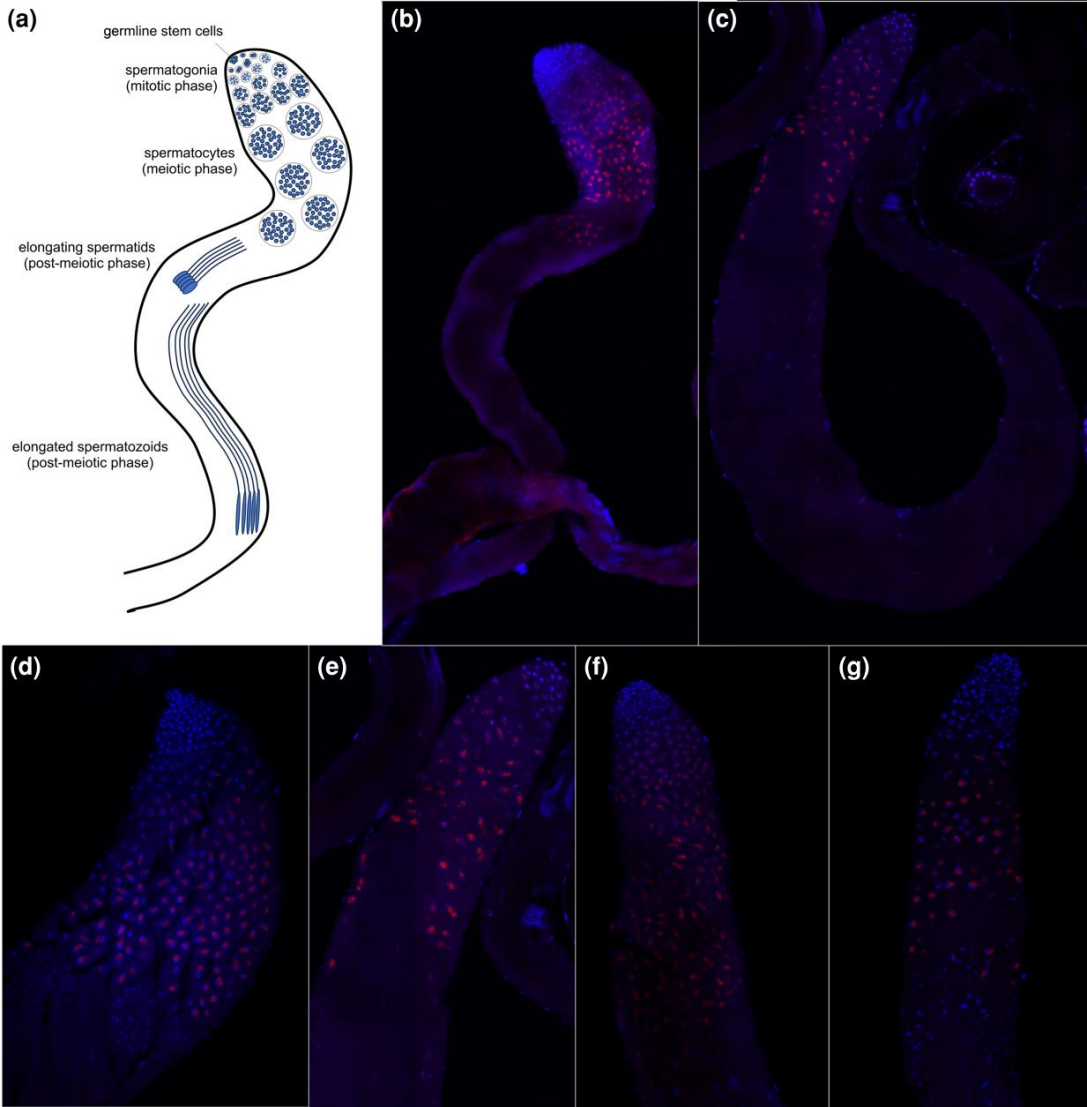
smRNA FISH of *OdsH* in the testes of *D. arizonae*, *D. mojavensis baja*, and its hybrids. a) Scheme of *Drosophila* spermatogenesis, based on Witt *et al.* (2019). b) Panorama of testes of *D. mojavensis baja*. c) Panorama of the testes of *D. arizonae*. d) Apical region of the testis of *D. mojavensis baja*. e) Apical region of the testis of *D. mojavensis baja*. f) Apical region of the H♀moj^baja^♂ari (fertile) testis. g) Apical region of the H♀ari♂moj^baja^ (sterile) testis. Notes—blue: DAPI; red: *OdsH* probes. H, hybrid.

To identify whether the expression of *OdsH* in the testis of sterile hybrids is atypical in other *Drosophila* groups, as described for the crosses between *D. mauritiana* and *D. simulans*, we analyzed species from the *repleta* group that show incipient speciation. We performed smRNA FISH of *OdsH* in the testes of *D. arizonae* and *D. mojavensis baja* species and their respective hybrids, since their hybrids present a sterile or fertile phenotype depending on the cross direction. During spermatogenesis, spermatocytes are known to show an increase in cell and nuclear volume and open chromatin (Fig. 6a). We observed *OdsH* transcripts in the primary and secondary spermatocytes in the parental strains (Fig. 6b–e). The patterns of the spermatocyte staining do not seem to be different from the parental ones in both H♀moj^baja^♂ari (fertile; Fig. 6f) and H♀ari♂moj^baja^ (sterile; Fig. 6g) hybrids. In addition, no signal of *OdsH* expression was observed in cells at the extreme apex of the testes or in the postmeiotic stages. Furthermore, we could observe that the sterile hybrids differ from the fertile ones by the defective formation of the sperm bundles (Supplementary Fig. 10).

To identify whether the expression of *OdsH* could be quantitatively differentiated in these hybrids, we quantified its expression in the testes of the parental species and their hybrids by qRT-PCR (Supplementary Table 9). The levels of expression were different (KW = 22.24, *P* < 0.001) between parental species but not between hybrids or between parental strains and hybrids, except for the comparison of *D. mojavensis baja* and H♀ari♂moj^baja^ (Supplementary Fig. 11).

## Discussion

### The emergence of a new duplicate in the Drosophilinae subfamily

The hypothesis of the *OdsH* origin from a duplication of the *unc-4* gene in the *Sophophora* subgenus ancestor was proposed by Ting *et al*. (2004). It was based on the presence of this gene in species of the *melanogaster* and *obscura* groups (*Sophophora* subgenus) without dating the duplication. To answer this question, we looked for sequences homologous to *unc-4* in all available genomes of the Drosophilidae (Bächli 2016). We identified *unc-4* duplicates in genomes from 6 genera of the Drosophilinae subfamily (*Drosophila*, *Scaptodrosophila*, *Chymomyza*, *Scaptomyza*, *Lordiphosa*, and *Zaprionus*) but not in Steganinae or other families of Diptera. This suggests that the duplication occurred much earlier than previously suggested by Ting *et al*. (2004) and placed the duplication in the ancestor of the subfamily Drosophilinae. We cannot exclude the possibility that the duplication occurred in a more basal node and was lost in other branches, but we do not have any argument to support this scenario. Also, no evidence of *unc-4* duplicates was observed in the genome of *D. erecta*, which might have lost *OdsH*. Loss of one of the copies due to accumulation of random mutations is a common fate among duplicated genes (Ohno 1970; Wolfe and Shields 1997; Inoue *et al*. 2015). Moreover, in *D. melanogaster*, the knockdown of this duplicate has no effect on the individual’s viability (Sun *et al*. 2004; Cheng *et al*. 2012).

Because the orthology of each duplicate and the paralogy between them are supported by the *in tandem* positioning in the assembled genomes (Fig. 1) and the phylogenetic relationships (Fig. 3), which are robust evidence of homology (Altenhoff *et al*. 2019), we considered that the duplicated gene is *OdsH*. By using a Bayesian phylogenetic inference approach, we conclude that *OdsH* and *unc-4* belong to sister monophyletic clades, which is evidence of a unique evolutionary origin of *OdsH* in Drosophilinae.

The presence of *OdsH* exclusively in Drosophilinae and in all its subgenera indicates that duplication occurred in the ancestral lineage of this subfamily at an estimated time of 62 MYA, right after the spread of the ancestor lineages of the subfamilies Steganinae/Drosophilinae. Suvorov *et al*. (2022), using genomic data, developed a broad dating analysis of Drosophilidae, whose divergences were estimated to be 63.2 MYA. The estimate for the divergence of the Drosophilinae subfamily in our analysis (53.3 MYA in the *unc-4* clade and 48.81 MYA in the *OdsH* clade) is close to that proposed by Suvorov *et al*. (2022) (53.4 MYA).

### *OdsH* and *unc-4*: same origin but divergent evolutionary histories

The sequences of *OdsH* and *unc-4* have evolved asymmetrically, since the former shows a higher divergence along Drosophilinae. *OdsH* shows more indels and thus smaller regions that can be aligned between the orthologous sequences in comparison to *unc-4* (Fig. 2). Moreover, *OdsH* showed higher rates of amino acid replacements and relaxation of negative selection than its paralog *unc-4* on the Drosophilinae ancestor. Along its divergence, we estimate stronger negative selection and selection homogeneity among species groups. These results are in agreement with the scenario of ancient neofunctionalization driven by positive selection right after the duplication, and that when a new function is established, the evolutionary rates decelerate under purifying selection, losing the signatures of ancient positive selection due to the saturation of synonymous substitutions (Van de Peer *et al*. 2001; Jordan *et al*. 2004; Dong *et al*. 2012; Pegueroles *et al*. 2013). Indeed, positive selection cannot be identified after 30–50 MYA, due to the accumulation of synonymous substitutions (Hughes 1999; Hughes *et al*. 2000).

In addition to sequence and phylogenetic divergence, we did not observe the presence of *unc-4* expression in the gonads of males (except for *D. yakuba* and *D. ananassae*) and females (Supplementary Fig. 9). *unc-4* is conserved in Metazoa, and its expression in the analyzed species is in agreement with the data observed for the single copy of the outgroup *T. dalmani* (Supplementary Fig. 9). This functional conservation is also supported by its lower diversity of putative TFBSs (Fig. 4a) and lack of amino acid replacements in its homeodomains and octapeptides in Drosophilinae when compared to the single-copy gene in Steganinae, indicating energy stability of homeodomain binding to DNA (Fig. 5).

Regarding *OdsH*, by using public data sets from NCBI, we observed expression exclusively in male reproductive tracts and testes in *Drosophila*, except for *D. pseudoobscura* (Supplementary Fig. 8). We also found that *OdsH* expression levels were higher (from 169.5 to 340 normalized read counts) than *unc-4* expression levels (less than 50 normalized read counts; Banho *et al*. 2021) in transcriptomes of the reproductive tracts from 2 *D. mojavensis* subspecies and *D. arizonae* previously sequenced by our group (BioProject NCBI PRJNA691040). Additionally, the expression levels of both genes in the female reproductive tract were lower than 10 counts (Banho *et al*. 2021).

In contrast to *unc-4*, the *OdsH* sequence was enriched in a greater diversity of TFBSs in its regulatory regions (Fig. 4a), which is in agreement with the observation of higher complexity in the regulatory regions of ancient daughter duplicates during their divergence (Zhang and Zhou 2019). In addition, TFBSs related to the development of the male reproductive system and to the initial stages of spermatogenesis (*achi*, *so*, and *vis*) were enriched in *OdsH*. It is known that *achi* and *vis* are expressed in primary spermatocytes, acting on the specification of the spermatogenesis gene regulation program (Ayyar *et al*. 2003; Wang and Mann 2003). Moreover, it has been shown that *so* is expressed in the cyst cells of the apical region of the *Drosophila* testis and contributes to the normal development of primary spermatocytes (Fabrizio *et al*. 2003).

Particularly with respect to sequence divergence, the OdsH protein shows greater divergence of the homeodomain than Unc-4, which can disturb the DNA binding energy, making the system more unstable (Fig. 5). These particularities of OdsH might make the binding of its homeodomain to its DNA target sites less specific than that of Unc-4. This suggests that the 2 proteins, which are transcription factors, have different binding sites in the target DNA that they regulate. However, OdsH, like Unc-4, has the conserved homeodomain amino acid Q47, which gives high cooperativity to homeodomains, with cooperativity being the main factor involved in the specificity of homeodomain binding to DNA target sites (Wilson *et al*. 1995). The amino acids that directly interact with the nitrogenous bases of DNA are also conserved in OdsH and Unc-4 (V44 and N48; Wilson *et al*. 1995), with the exception of *D. mauritiana*, which has an isoleucine at residue 44 of the OdsH homeodomain.

In view of the evolutionary changes discussed above, we propose that neofunctionalization of *OdsH* occurred in the testes of the Drosophilinae ancestor. *OdsH* seems to have evolved different functions subsequently in the Drosophilinae evolutionary lineages, since it is expressed in the reproductive tract besides the testis in *Drosophila*. In addition, we found no *OdsH* expression in the testis of *D. persimilis* and *D. virilis*, but it is expressed in their male reproductive tract, as well as in male and female head and testis of *D. pseudoobscura*. Our findings are in agreement with previous reports of new function acquisition by newly duplicated genes in *Drosophila* testis (Betrán *et al*. 2002; Zhang *et al*. 2010; Assis and Bachtrog 2013; Assis 2014; Chakraborty and Fry 2015; Jiang and Assis 2017) and with the out-of-testis hypothesis (Kaessmann 2010).

The dating of the duplication that originated *OdsH* at 62 MYA and our hypothesis of early neofunctionalization finds support in Bao *et al*. (2018), who demonstrated that duplicates in *Drosophila*, dated to approximately 60 MYA, underwent higher rates of neofunctionalization and innovative evolution. This may have configured a propitious scenario for fixing substitutions and neofunctionalization at the time of *OdsH*/*unc-4* duplication.

### The role of *OdsH* in the hybrid sterility

Regarding faster evolution as a source of incompatibility between hybridizing genomes, the signatures of negative selection were homogeneous in the Drosophilinae, except in the *melanogaster* complex (Table 1) and in the *immigrans* group (Supplementary Table 6). However, our analyses did not identify positive selection in the *melanogaster* complex as in the pairwise analysis reported by Ting *et al*. (1998). Regarding the positive selection in the *immigrans* group, none of the sites presenting signatures of positive selection are within the homeodomain but in its C-terminal tail. Therefore, none of them interact directly with the DNA strand, but they could still cause variations in the protein stability or cooperation with other cofactors.

Our protein sequence analysis identified the replacement of the amino acid valine, conserved at site 44, which interacts directly with nitrogenous bases of its binding site in DNA, by isoleucine in the *OdsH* of *D. mauritiana* (Fig. 5c). This might change the binding sites on the genome. It was previously identified that *D. mauritiana OdsH* binds to the heterochromatic region of the Y chromosome, whereas that of *D. simulans* does not bind to this region (Bayes and Malik 2009), which may be caused by this difference in the DNA strand–binding amino acid in *D. mauritiana*. Moreover, the OdsH proteins in the 2 species are the ones with the highest values of DNA-binding instability (Fig. 5c), probably driven by the relaxation of negative selection observed in these sequences.

Additionally, the specificity of binding to sites on the DNA strand depends mainly on transcription cofactors that act linked to homeodomains (Wilson *et al*. 1995; Bürglin and Affolter 2016). The evolution of homeodomains of the paired-like phylogenetic class, to which Unc-4 (Winnier *et al*. 1999) and *OdsH* belong, occurs through rearrangements and losses of functional motifs, including the octapeptide. The diversity of protein structures in this class of proteins is mainly related to the presence/absence of functional motifs between its families (Jacob 1977). Indeed, the presence of the octapeptide is conserved in Unc-4 of *C. elegans* and binds to the transcription cofactor Unc-37 (orthologous to Groucho, in *Drosophila*), repressing its target expression (Winnier *et al*. 1999).

Our analyses also showed that OdsH of *D. mauritiana* does not show the octapeptide, which is conserved at the C-terminal ends of Unc-4 and OdsH of the other Drosophilinae. Since the *OdsH* molecular mechanism of action occurs through the interaction of different loci (Bayes and Malik 2009; Lu *et al*. 2010), the structural features of the OdsH protein from *D. mauritiana* might result in incompatibility within the *D. simulans* genome, as proposed by the Dobzhansky–Muller model (Dobzhansky 1937; Muller 1942). This incompatibility leads to the phenotype of defective sperm bundle formation, resulting in immobility (Lu *et al*. 2010).

We previously observed sperm immobility in sterile hybrids of *D. arizonae*–*D. mojavensis* (Banho *et al*. 2021), and defects in sperm bundles have been observed (Supplementary Fig. 10; Hardy *et al*. 2011; Kanippayoor *et al*. 2020), as observed also in hybrids from *D. mauritiana* and *D. simulans* (Perez *et al*. 1993). In these species, we showed that *OdsH* expression occurs during the differentiation of spermatocytes (Fig. 6), in which intensive cell growth and greater synthetic RNA activity occur (Hackstein 1987). In addition, for these species, the nucleus of mature primary spermatocytes has been described as dumbbell shaped (Pantazidis *et al*. 1992), in which we can observe the highest intensity of *OdsH* probes (Fig. 6). Thus, our results indicate that *OdsH* is expressed in spermatocytes, as previously demonstrated in *D. simulans*, and its expression occurs in spermatocytes beginning in the G2 phase (Bayes and Malik 2009). However, this feature is observed in a reduced number of old gene duplicates, such as *OdsH*, which are mostly expressed in the mitotic phases of spermatogenesis (Raices *et al*. 2019; Su *et al*. 2021).

In contrast to the atypical intense expression of *OdsH* in the apical cells of the testes in the sterile offspring from *D. mauritiana* and *D. simulans* (Sun *et al*. 2004), we showed that the expression of *OdsH* in *D. arizonae*, *D. mojavensis baja*, and their sterile and fertile hybrids did not differ (Fig. 6; Supplementary Fig. 10). Since this gene is highly expressed premeiotic phase in sterile hybrids of *D. mauritiana* and *D. simulans*, contrary to parental species and fertile hybrids (Sun *et al*. 2004), our results could indicate that *OdsH* deregulation might not play a sterilizing role in hybrids of *D. mojavensis* and *D. arizonae*. Indeed, speciation genes have been characterized as lineage specific (Gomes and Civetta 2014), and *OdsH* might act as a speciation gene only in *D. mauritiana* and *D. simulans*. However, *OdsH* could still play some role in the molecular pathway of male fertility for *D. mojavensis* and *D. arizonae*, since they differ in their enriched homeodomain-containing TFBSs, including *so*, related to spermatocyte development (Fabrizio *et al*. 2003), as *D. simulans* and *D. mauritiana* (Fig. 4b).

In conclusion, we show here an older origin of *OdsH* than previously reported and the evolutionary process this duplicate underwent in Drosophilinae, as it evolved asymmetrically in relation to its ancestor gene *unc-4*. Since it presents innovative expression in the testes in *Drosophila* that was not observed for paralog and single copy *unc-4*, we propose that it went through neofunctionalization rapidly after its origin. We also report specific features that indicate protein divergence, particularly in *D. mauritiana*, which may be associated with the incompatibility described in introgression of this gene in the *D. simulans* genomic background. Our data show that even though it is the first speciation gene described in *Drosophila*, much of the evolutionary history that led *OdsH* to play a role in reproduction remains unknown and that its role as a speciation gene may be restricted to specific groups of species. The extent of such a role in the family Drosophilinae can only be determined with extensive studies using interspecific hybrids of closely related species similar to ours.

## Supplementary Material

jkad299_Supplementary_Data

## Data Availability

All data generated in this study are included in the supplementary information files. Supplemental material available at G3 online.
