# Supplementary material for: A comprehensive evolutionary scenario for the origin and neofunctionalization of the Drosophila speciation gene Odysseus (OdsH)
Source: G3 (Bethesda). 2023 Dec 29;14(3):jkad299. doi: 10.1093/g3journal/jkad299 (PMC10917504; doi:10.1093/g3journal/jkad299)
Supplement: jkad299_Supplementary_Data [file jkad299_supplementary_data.zip › Supplemental_Figures_G3-2023-404724.pdf]

SUPPLEMENTARY FIGURES

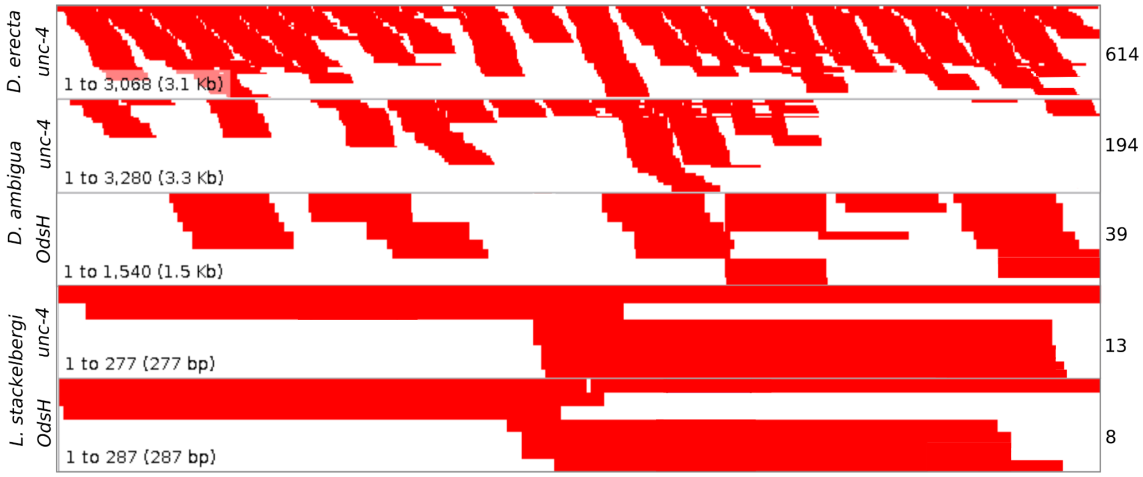

Supplementary Fig. S1. Genomic reads alignment on *unc-4* and *OdsH* in *D. erecta*, *D. ambigua* and *L. stackelbergi*. The reads have the length of 150pb. NOTES: The base pairs length of each sequence reference for the alignment is shown on the bottom left of each alignment and the number of aligned reads, on the right. *D. erecta* did not have any read aligned on *OdsH.*

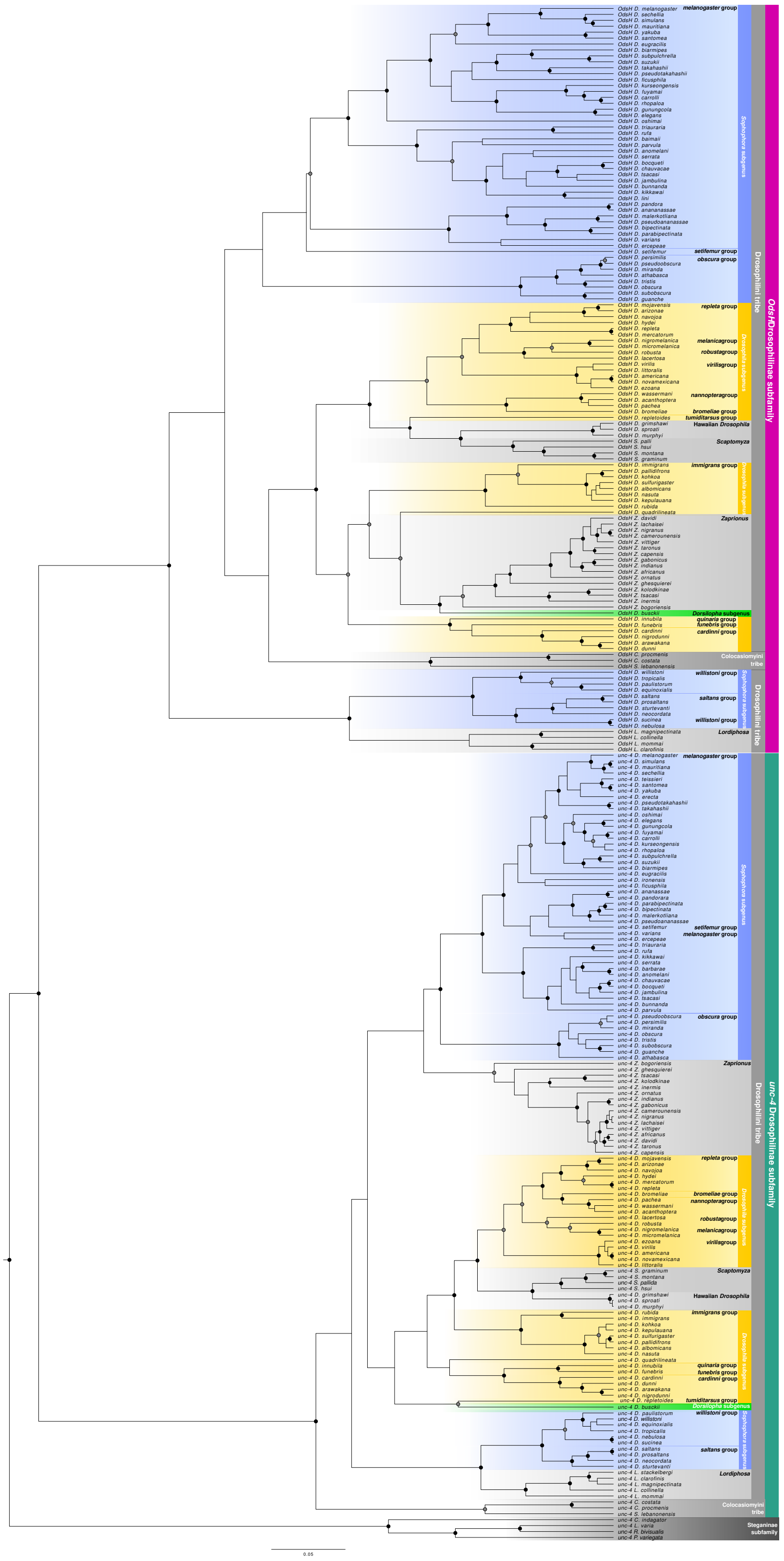

Supplementary Fig. S2. Bayesian inference of the phylogenetic relationships between the sequences of the paralogue genes *unc-4* and *OdsH*, using the GTR+G+I model of nucleotide substitution. The analysis was performed with 405 sites from 259 nucleotide sequences. All positions containing gaps and ambiguous positions were removed from the pairwise sequence analysis. At the root of each clade, the posterior probability is presented by black (>0.9) and grey (>0.7) circles and the estimated time of divergence are indicated. The analysis was conducted in BEAST v16.1. The *unc-4* clade, subdivided into more basal single copy Steganinae (outgroup) and Drosophilinae, is presented at the base of the phylogeny followed by the *OdsH* clade in the upper part. Subgenera are highlighted in blue (*Sophophora*), yellow (*Drosophila*) and green (*Dorsilopha*).

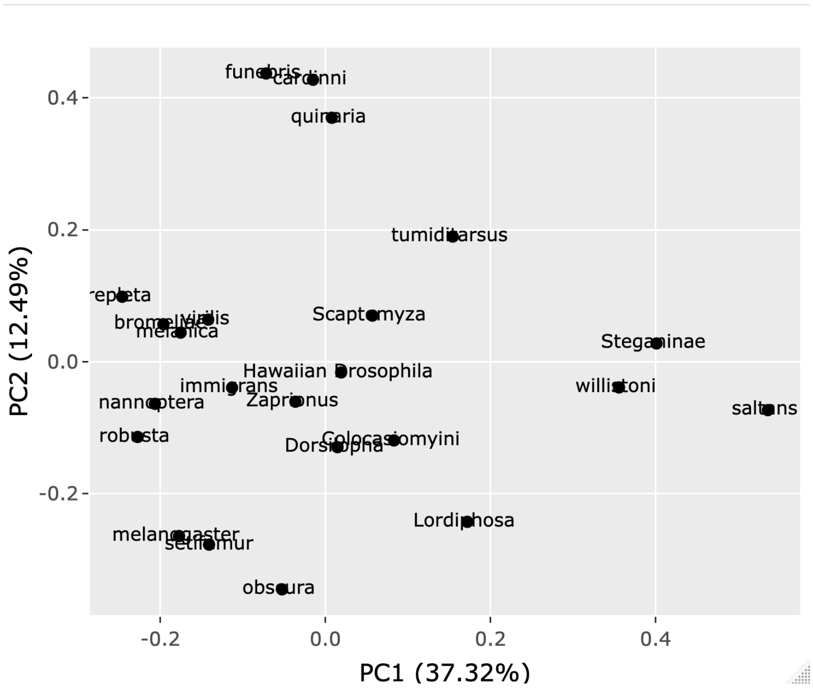

Supplementary Fig. S3. PCA of the codon usage estimated by the RSCU calculation of *unc-4* sequences of Drosophilinae.

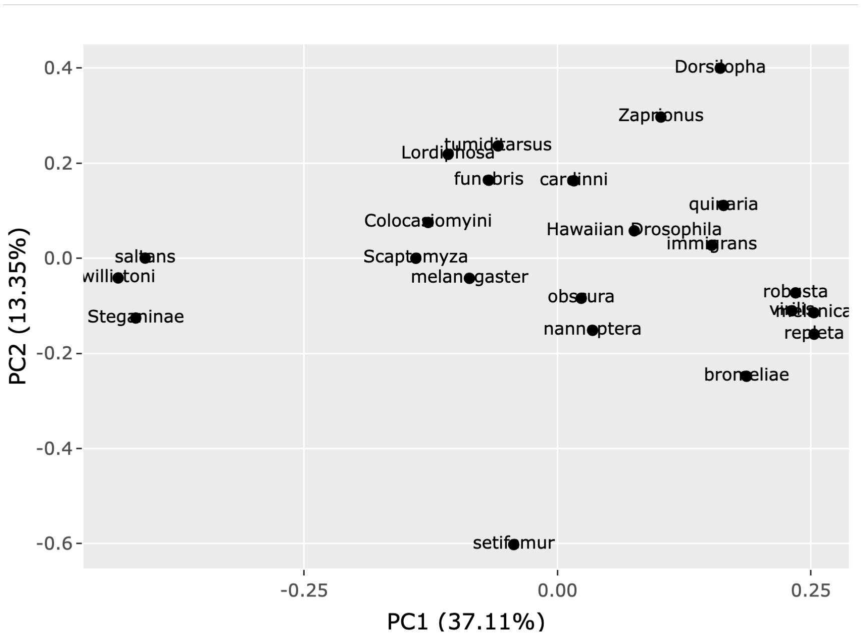

Supplementary Fig. S4. PCA of the codon usage estimated by the RSCU calculation of *OdsH* sequences of Drosophilinae and the orthologue single copy *unc-4* in Steganinae.

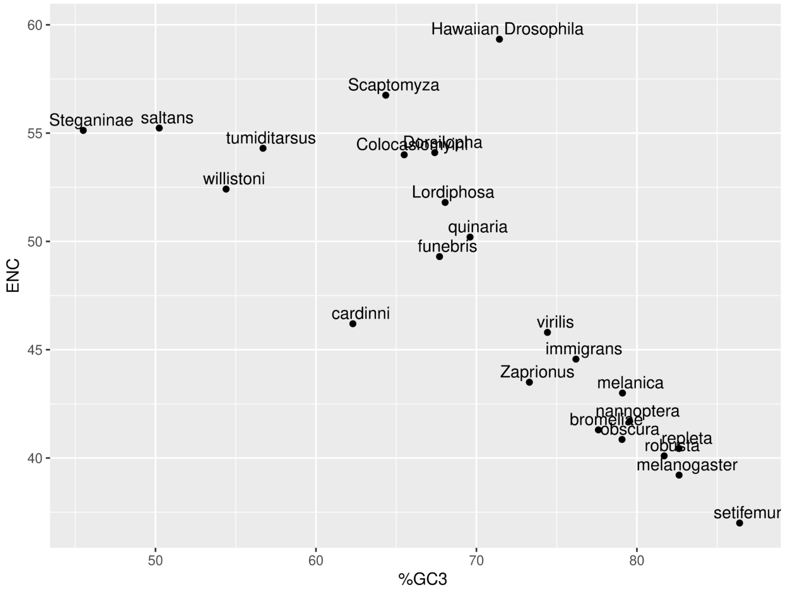

Supplementary Fig. S5. % of GC3 and ENC in Drosophilidae *unc-4* sequences.

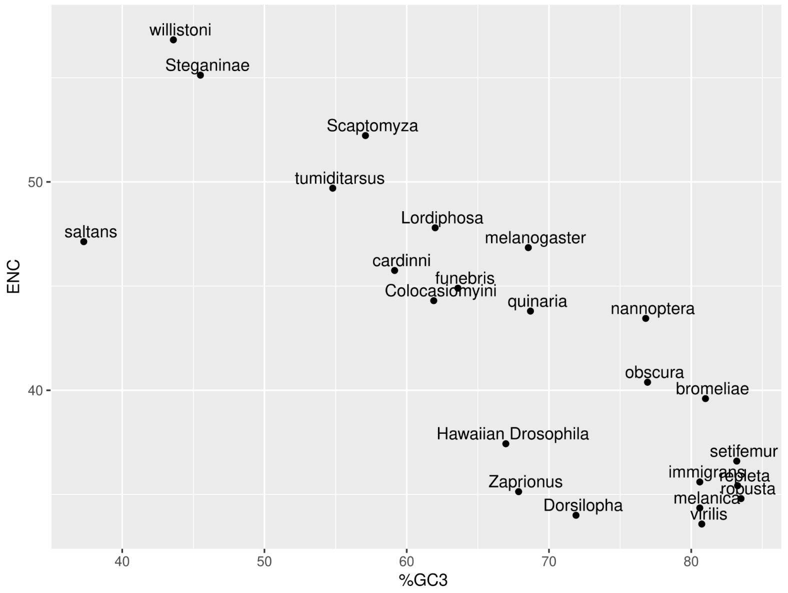

Supplementary Fig. S6. % of GC3 and ENC in *OdsH* sequences of Drosophilinae and the orthologue single copy *unc-4* in Steganinae.

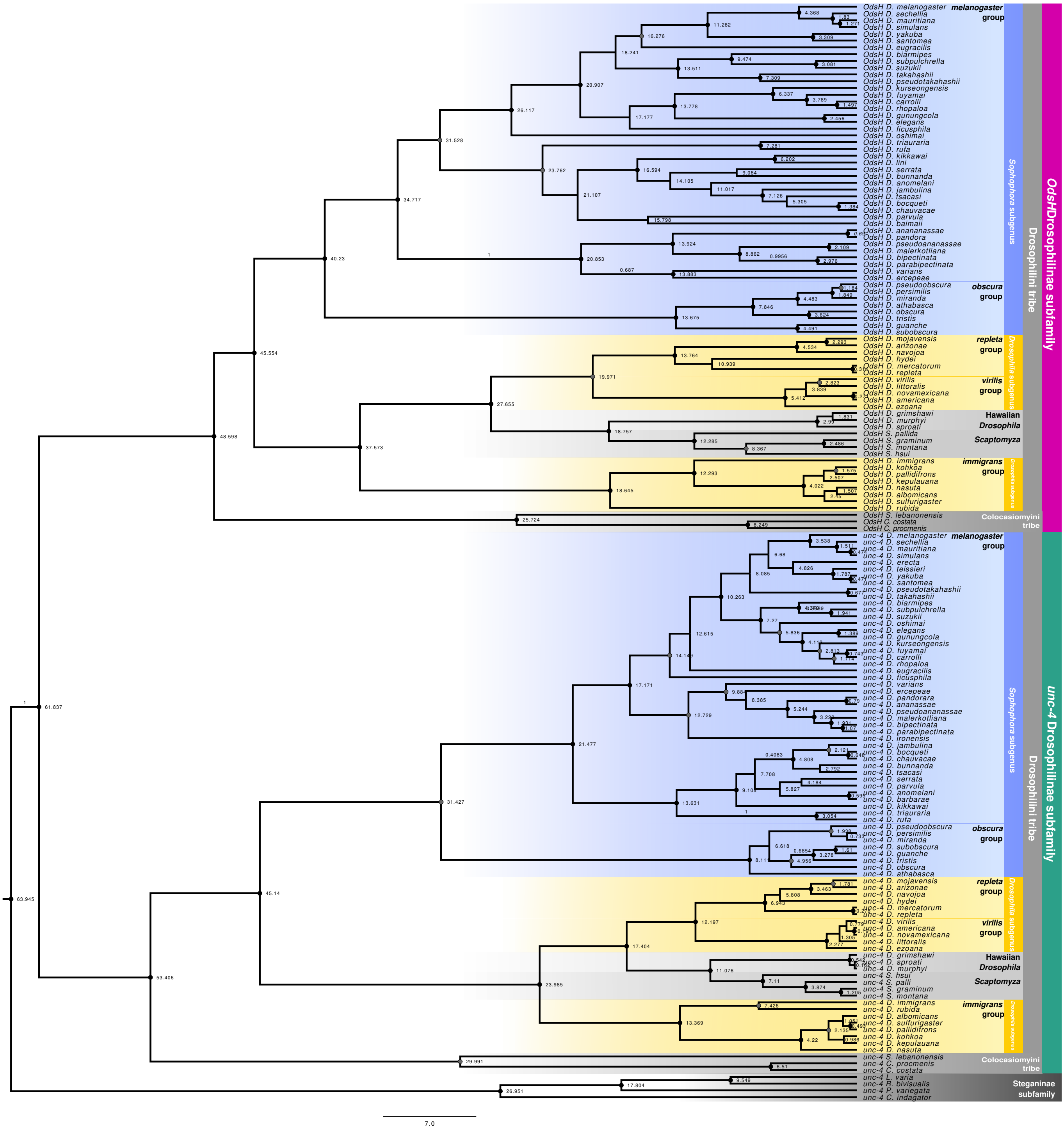

Supplementary Fig. S7. Calibrated Bayesian phylogenetic inference of the sequences of the paralogue genes *unc-4* and *OdsH*, using the GTR+G+I model of nucleotide substitution. The analysis was performed with 405 nucleotide sites from 162 nucleotide sequences. All positions containing gaps and ambiguous bases were removed from the pairwise sequence analysis. The branches referring to the *Drosophila* taxonomic groups were compressed. At the root of each clade, the posterior probability is presented by black (>0.9) and grey (>0.7) circles and the estimated time of divergence are indicated. The analysis was conducted in BEAST v16.1. The *unc-4* clade, subdivided into more basal single copy Steganinae (outgroup) and Drosophilinae, is presented at the base of the phylogeny followed by the *OdsH* clade in the upper part. Subgenera are highlighted in blue (*Sophophora*) and yellow (*Drosophila*).

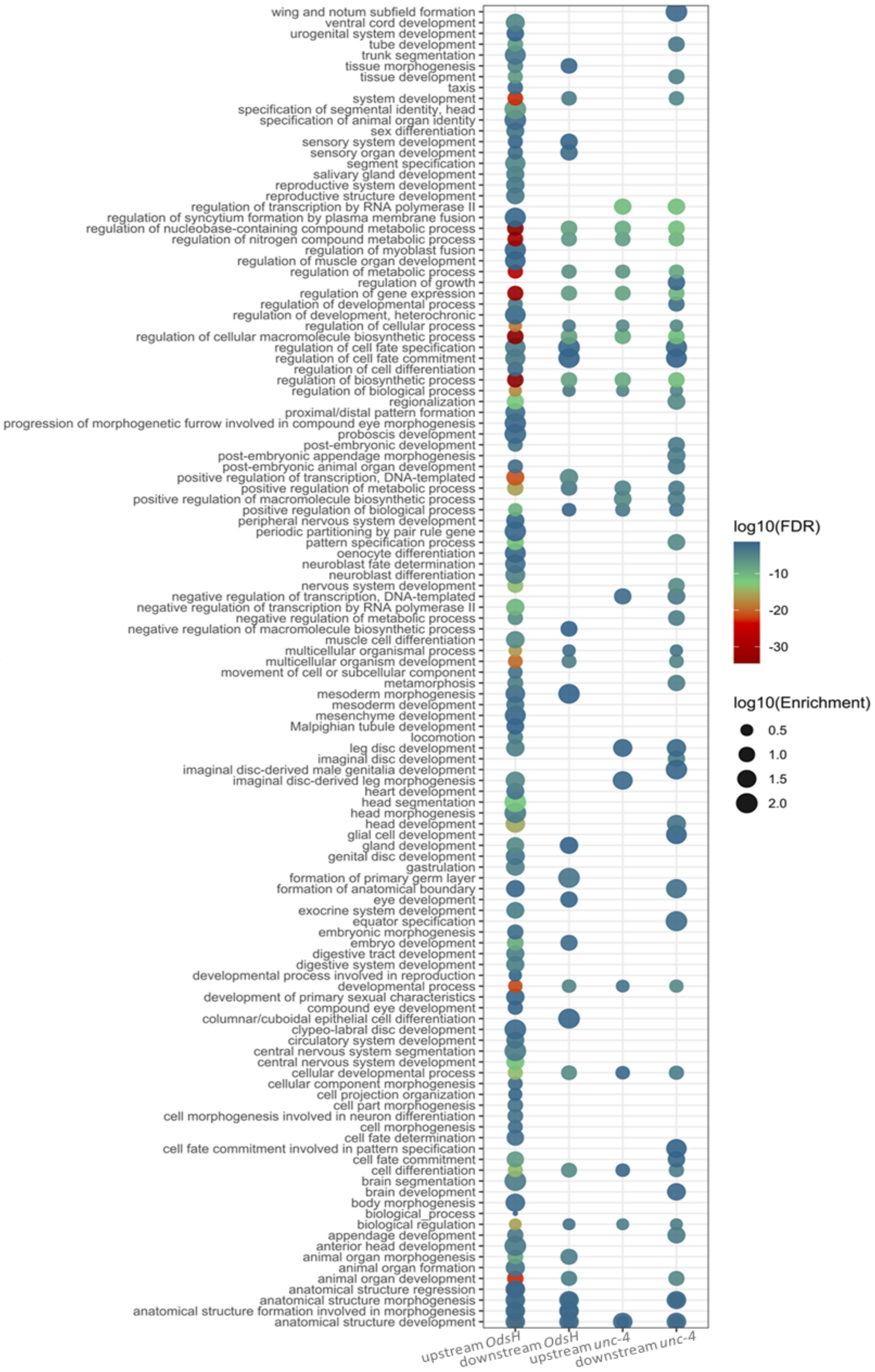

Supplementary Fig. S8. Enriched GO terms for biological process category of TFBS in the regulatory region of *OdsH* and *unc-4* of *Drosophila.*

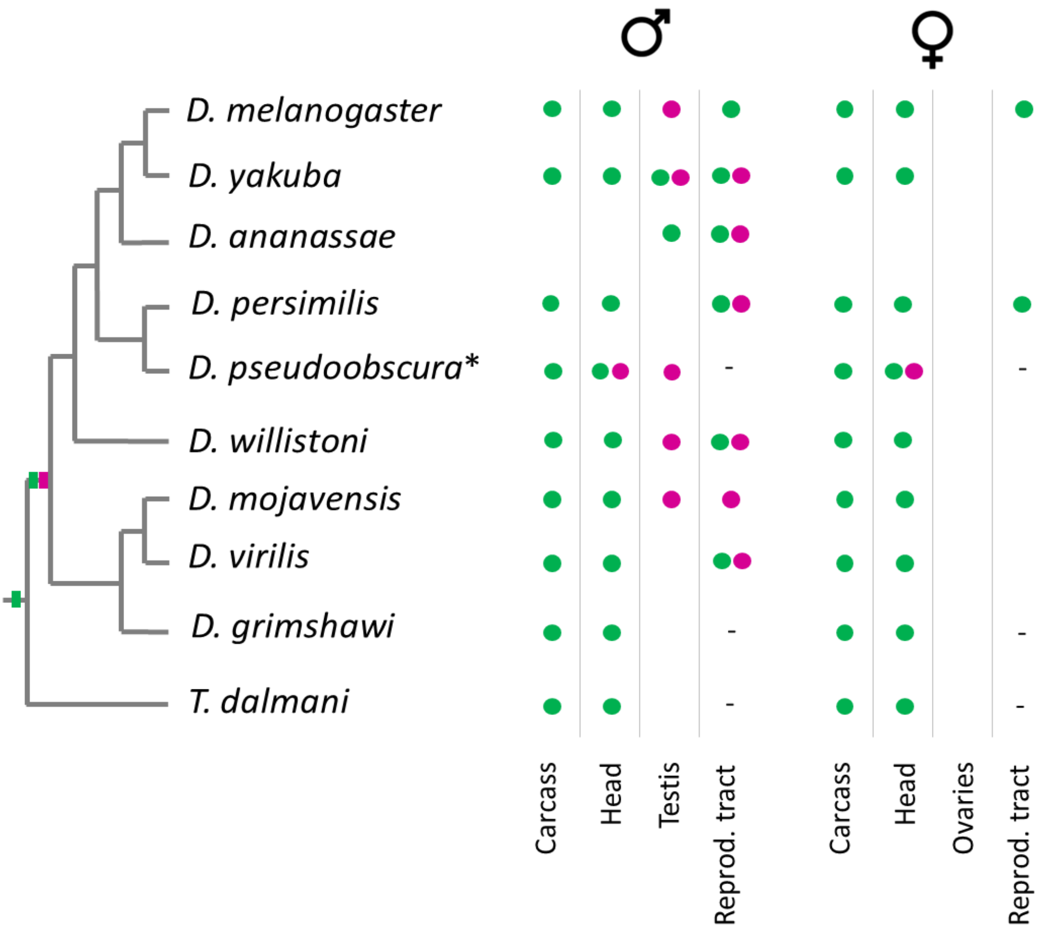

Supplementary Fig. S9. Expression of *OdsH* (pink) and *unc-4* (green) in each tissue of *Drosophila.* The representation of the phylogenetic relationships of the species was based on Suvorov et al. (2022). The squares on phylogeny represent the presence of the genes *OdsH* (pink) and *unc-4* (green). *Teleopsis dalmani* is the outgroup that presents *unc-4* single copy.

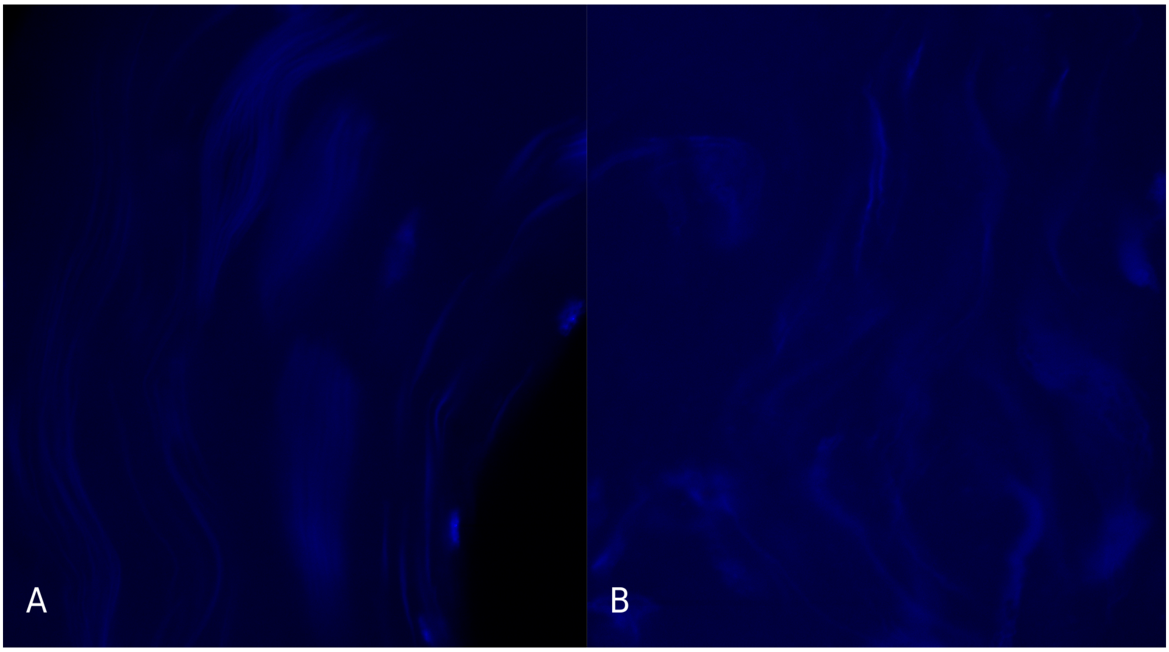

Supplementary Fig. S10. Sperm bundles in hybrids from *D. m. baja* and *D. arizonae*. A. H♀moj^{baja}♂ari (fertile). B. H♀ari♂moj^{baja} (sterile). NOTE: DAPI (blue) was used to label DNA.

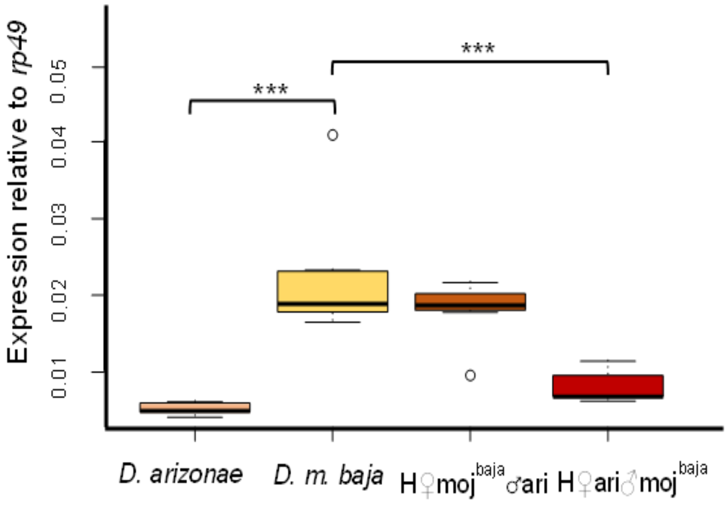

Supplementary Fig. S11. Relative expression of *OdsH*, in relation to *rp49*, in tissues *D. arizonae* and *D. m. baja* and their reciprocal hybrids. NOTE: *** $p<0.01$
